# The PR-Set7 binding domain of Riz1 is required for the H4K20me1-H3K9me1 *trans*-tail ‘histone code’ and Riz1 tumor suppressor function

**DOI:** 10.1093/nar/gkt1377

**Published:** 2014-01-13

**Authors:** Lauren M. Congdon, Jennifer K. Sims, Creighton T. Tuzon, Judd C. Rice

**Affiliations:** Department of Biochemistry and Molecular Biology, University of Southern California Keck School of Medicine, Harlyne J. Norris Cancer Research Tower, Los Angeles, CA 90033, USA

## Abstract

PR-Set7/Set8/KMT5a is the sole histone H4 lysine 20 monomethyltransferase (H4K20me1) in metazoans and is essential for proper cell division and genomic stability. We unexpectedly discovered that normal cellular levels of monomethylated histone H3 lysine 9 (H3K9me1) were also dependent on PR-Set7, but independent of its catalytic activity. This observation suggested that PR-Set7 interacts with an H3K9 monomethyltransferase to establish the previously reported H4K20me1-H3K9me1 *trans*-tail ‘histone code’. Here we show that PR-Set7 specifically and directly binds the C-terminus of the Riz1/PRDM2/KMT8 tumor suppressor and demonstrate that the N-terminal PR/SET domain of Riz1 preferentially monomethylates H3K9. The PR-Set7 binding domain was required for Riz1 nuclear localization and maintenance of the H4K20me1-H3K9me1 *trans*-tail ‘histone code’. Although Riz1 can function as a repressor, Riz1/H3K9me1 was dispensable for the repression of genes regulated by PR-Set7/H4K20me1. Frameshift mutations resulting in a truncated Riz1 incapable of binding PR-Set7 occur frequently in various aggressive cancers. In these cancer cells, expression of wild-type Riz1 restored tumor suppression by decreasing proliferation and increasing apoptosis. These phenotypes were not observed in cells expressing either the Riz1 PR/SET domain or PR-Set7 binding domain indicating that Riz1 methyltransferase activity and PR-Set7 binding domain are both essential for Riz1 tumor suppressor function.

## INTRODUCTION

Compaction of the large eukaryotic genome in the small volume of the nucleus is facilitated by the formation of chromatin, a structure composed mainly of DNA and the canonical histone proteins. In addition to genome compaction, histones directly participate in the regulation of essential DNA-templated processes including transcription, replication and repair. The multitude of well-documented post-translational modifications known to occur on histones is one mechanism that mediates their diverse, yet specific, functional roles in DNA regulation. Increasing evidence supports the ‘histone code' hypothesis where a single or specific combination of histone post-translational modifications functions to establish and maintain the associated DNA-templated process at certain genomic loci (1). For example, the concurrent H3K4me3 and H3K27me3 modifications at promoters of developmentally regulated genes are associated with their transcriptionally ‘poised’ state in embryonic stem cells (2). During differentiation, these modifications separately resolve such that promoters of activated genes have only H3K4me3, whereas promoters of repressed genes have only H3K27me3.

In previous studies, we discovered a *trans*-tail ‘histone code’ involving the monomethylation of H4K20 and H3K9. We showed that affinity-purified H4K20me1-nucleosomes from human cells are selectively enriched with H3K9me1 and both modifications coincide at specific genomic regions (3). RNAi-mediated depletion of the PR-Set7/Set8/KMT5a H4K20 monomethyltransferase resulted in the expected ablation of H4K20me1 but also specific and significant reductions in global and local H3K9me1 (4). Because the reductions in H3K9me1 were found to be independent of PR-Set7 catalytic activity, we hypothesized that PR-Set7 interacts with an H3K9 monomethyltransferase to establish the H4K20me1-H3K9me1 *trans*-tail ‘histone code’. In this report, we demonstrate that PR-Set7 specifically and directly binds the Riz1/PRDM2/KMT8 tumor suppressor protein, which we determined to be an H3K9 monomethyltransferase responsible for a significant portion of global H3K9me1. In addition, we found that the PR-Set7 binding domain of Riz1 was required for Riz1 nuclear localization and the genomic maintenance of the H4K20me1-H3K9me1 *trans*-tail ‘histone code’. Furthermore, we demonstrate that Riz1 catalytic activity and the PR-Set7 binding domain are both required to restore Riz1 tumor suppressor function in Riz1-deficient cancer cells.

## MATERIALS AND METHODS

### Cell culture, transfections and immunoprecipitations

HeLa, U2OS, HEK293 and HCT116 cell lines (ATCC) were maintained in Dulbecco’s modified Eagle’s medium supplemented with 10% fetal bovine serum, 0.1 mM MEM non-essential amino acids, 2 mM l-glutamine and 1% Pen-Strep. Sf9 cells (Invitrogen) were maintained according to the manufacturer’s instructions. Transfections were performed using BioT (Bioland Scientific) according to the manufacturer’s instructions. Nuclei were isolated 24 h after transfection with nuclear isolation buffer [10 mM Hepes, pH 7.5, 1.5 mM MgCl_2_, 10 mM KCl, 0.5% NP-40, 0.5 mM dithiothreitol (DTT), 1 mM phenylmethylsulfonyl fluoride and protease inhibitors] and centrifugation. The nuclear pellet was re-suspended in IP lysis buffer (50 mM Tris–HCl, pH 7.0, 150 mM NaCl, 0.5 mM DTT and 1% NP-40) and incubated with either HA- or FLAG-conjugated beads before extensive washing and elution of the bound material with 30 µl 6× sodium dodecyl sulphate dye and boiling or FLAG peptide, respectively.

### Antibodies

**Figure 1.**
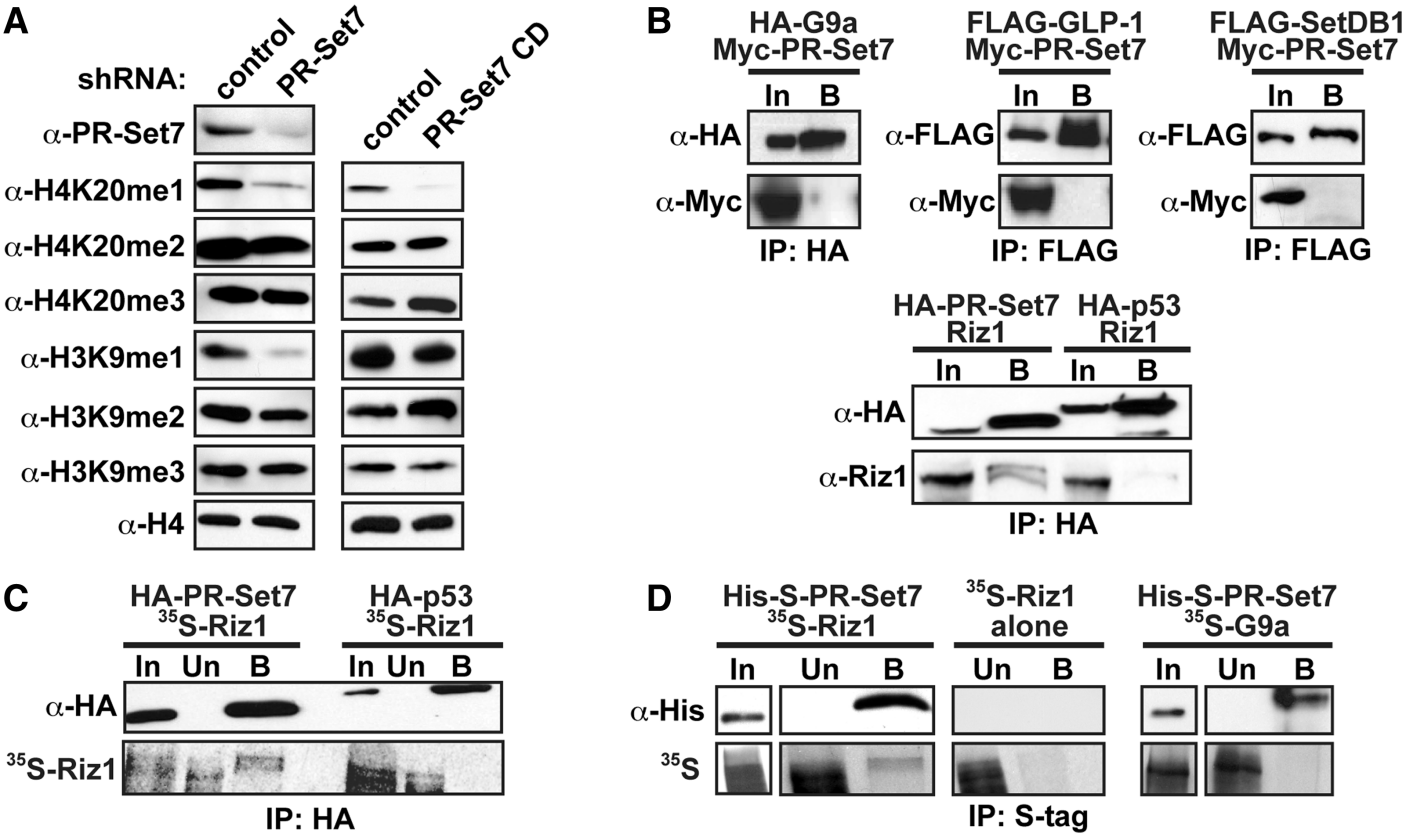
PR-Set7 selectively and directly binds Riz1. (**A**) Western analysis using indicated antibodies on HeLa whole cell lysates transfected with control or PR-Set7 shRNA (left), or a control or catalytically dead PR-Set7 (CD) plasmid (right). (**B**) HeLa cells co-transfected with the indicated plasmids were immunoprecipitated (FLAG or HA) followed by Western analysis of bound proteins. (**C**) HA-immunoprecipitations of HeLa nuclear extracts expressing HA-PR-Set7 or HA-p53 incubated with *in vitro* translated ^35^S-Riz1 were fractionated by SDS-PAGE before Western analysis (top) or autoradiography (bottom). (**D**) S-tag-immunoprecipitations of recombinant His-S-PR-Set7 incubated *in vitro* with ^35^S-Riz1 or ^35^S-G9a. Bound material were fractionated by SDS-PAGE before Western analysis (top) or autoradiography (bottom).

Western analysis was performed using the following antibodies and dilutions: PR-Set7 (Cell Signaling; 1:1K), H4K20 methyl-specific (Active Motif; 1:5K), H3K9 methyl-specific (Millipore; 1:10K), H4 (Abcam; 1:60K), H3 (Abcam; 1:100K), HA (Roche; 1:1K), Myc (Roche; 1:1K), FLAG (Sigma; 1:2.5K), Riz1 [Abcam ab3790 (Figure 1) or ab9710 (Figures 3 and 6); 1:250], His (Novagen; 1:1K), S-tag (Abcam; 1:1K), GST (Millipore; 1:5K), Gal4-DBD (Santa Cruz; 1:5K), G9a (Millipore; 1:1K) and HP1β (Millipore; 1:5K).

### Expression analysis by quantitative real-time polymerase chain reaction

Total RNA was isolated using the RNeasy Mini Kit (Qiagen) and converted to cDNA using TaqMan Reverse Transcription Reagents Kit (Applied Biosystems) as previously described (5). Quantitative real-time polymerase chain reaction (RT-qPCR) was performed with SYBR Green (Bio-Rad) using a Bio-Rad I-Cycler. Expression was normalized to *18S* expression, and three independent biological replicates were performed to generate standard deviation. The Student’s *t* test was used to determine statistical significance. Primer sequences used for amplifications were as follows (forward, reverse): *RUNX1* (5′-ACTTCCTCTGCTCCGTGCT, 5′-GCGGTAGCATTTCTCAGCTC); *BRD1* (5′-ATGAAGGCTGCCAAAGAGAA, 5′-TTTCCTCATTGTGGCAAAGTC); *ADM* (5′-GGACGTCTGAGACTTTCTCCTT, 5′-ACGACTCAGAGCCCACTTATTC); *NFKBIZ* (5′-TGGTTGATACCATTAAGTGCCTA, 5′-GTAAGCCTTTGCATTCACAAAA); *VAMP1* (5′-AGCATCACAATTTGAGAGCAGT, 5′-GTGTTGAGAGAGCAAACAGAGG); *ARL14* (5′-AGAACTGTTTGGGGCTGTTACT, 5′-CACTGCAAAGCTTCTTCACTTT); *HES5* (5′-AGCTACCTGAAGCACAGCAA, 5′-GAAGTGGTACAGCAGCTTCATC); *REC8L1* (5′-AATTCCAGGAACAACTGCAAA, 5′-TTGGAACTTCAATCTTTCTCCTT); *CBR1* (5′-TGATCCCACACCCTTTCATA, 5′-AGCTTTTAAGGGCTCTGACG); *TUG1* (5′-ACCAAGGAGTCCCCTTACCT, 5′-GCCTTTGGAAAACCAATGAT); *18S* (5′-AACTTTCGATGGTAGTCGCCG, 5′-CCTTGGATGTGGTAGCCGTTT); *PR-Set7* (5′-ATTGCCACCAAGCAGTTCTC, 5′-CGATGAGGATGAGGTGAGGT); and *Riz1* (5′-AATTTGGGATGGATGTGCATTG, 5′-GGCGCGATTGGCTTTAAAGT-3′).

### Plasmids and recombinant proteins

The pSUPERIOR.retro.puro control and PR-Set7 small hairpin RNAs (shRNAs) and pQCXIP-PR-Set7 plasmids were described previously (6). Human Riz1 shRNA (5′-AACTGGCTGCGATATGTGAAT) was inserted into pSUPERIOR.retro.puro (OligoEngine). FLAG-HA-Riz1 was inserted into pcDNA4/TO (Invitrogen) for transfections. The pmCherry-C1 plasmid was engineered by replacing EGFP of pEGFP-C1 (Clontech) with mCherry before inserting Riz1. For His-S-tag recombinant protein purification, PR-Set7 was inserted into pET-45b(+) (Novagen), transformed into BL21 *Escherichia coli*, grown until OD ∼0.6–0.8, placed at 4°C for 1 h, induced with 0.5 mM IPTG at 16°C overnight, affinity-purified using Ni-Sepharose high-performance agarose beads (GE Healthcare) and eluted with 400 mM imidazole. For GST recombinant protein purification, Riz1 was inserted into pGEX-4T-1 (GE), transformed into BL21 *E. coli*, grown until OD ∼0.6-0.8, induced with 0.2 mM IPTG at 37°C for 5 h, affinity-purified using Glutathione Sepharose 4B beads (GE) and eluted with 10 mM reduced glutathione in 50 mM Tris–HCl, pH 8.0. The Bac-to-Bac Baculovirus Expression System (Invitrogen) was used to produce recombinant pFastBacHT-B bacmids. Sf9 cells were transfected with recombinant bacmid, incubated at 27°C for 5 days and viral supernatant was isolated. High titer P2 viral stock was obtained after two rounds of amplification (5 days each) for large-scale transduction of Sf9 cells.

### *In vitro* histone methyltransferase assays

Assays were performed as previously described (7). Briefly, reactions (30–60 µl) containing 20 mM Tris-HCl, pH 8.0, 0.2 M NaCl, 0.4 mM EDTA, 1 mM DTT, 3 µg affinity-purified GST-fusion proteins, 1 µg H3 peptides and 3 µl [methyl-^3^H] SAM (Perkin Elmer) were incubated at 37°C for 1.5 h, spotted on P-81 filter paper, washed and measured by scintillation counting.

### Microscopy and flow cytometry

U2OS or HeLa cells on glass coverslips were transfected with mCherry Riz1 and 24 h later were counterstained with 4′,6-diamidino-2-phenylindole (DAPI) before visualization using the 63× objective of a Zeiss Axio Imager. Images were analyzed using Adobe PhotoShop CS6. HCT116 cells transfected with mCherry plasmids were harvested 4 days later and stained for DNA content with Hoechst 33342. Flow cytometry was performed to analyze the DNA content of mCherry positive cells using a BD FACSAria cell sorter fitted with a 70-µm nozzle.

### ChIPs and qPCR

ChIPs were performed using α-H4K20me1, α-H3K9me1 (Active Motif) or α-H3 (Abcam) antibodies as previously described (4). Briefly, 0.1 ng of ChIPed DNA was used as template in qPCR reactions with primers designed to amplify *RUNX1* (5′-GGTGGAGGAGTATCGTCTCG, 5′-ATCTGTGAACTCGGGTTTGG), *BRD1* (5′-TACCTGGTCCTATCGGATGC, 5′-AAGACTGTCCCCTCAGACCA) or *CYYR1* (5′-CTCTCCCTTCGCTCCAAGC, 5′-TCACTGACCTGCGTAGACAAAGA). Results were plotted as the fold enrichment of the percentage input of IP relative to the percentage input of H3 general ChIP. HEK293 TK22 cells (8) were used for ChIPs as previously described (9).

## RESULTS

### PR-Set7 specifically and directly binds the Riz1 H3K9 methyltransferase

RNAi-mediated depletion of PR-Set7 in HeLa cells resulted in the expected reduction of global H4K20me1 but also an unexpected reduction of H3K9me1, as previously reported (Figure 1A) (4). Cellular H3K9me1 levels are not dependent on PR-Set7 enzymatic activity, as ectopic expression of a dominant negative PR-Set7 catalytically dead point mutant significantly reduced global H4K20me1 but not H3K9me1 (Figure 1A, Supplementary Figure S1). Taken together, these findings strongly suggested that PR-Set7 interacts with an unknown H3K9 monomethyltransferase and that this interaction is required for a significant portion of H3K9me1 in human cells. To identify this enzyme, HeLa cells were co-transfected with a Myc-PR-Set7 plasmid and epitope-tagged plasmids of three different well-characterized H3K9 methyltransferases: G9a, GLP-1 and SetDB1 (10,11). Western analysis following immunoprecipitation of each tagged H3K9 methyltransferase demonstrated that PR-Set7 does not interact with G9a, GLP-1 or SETDB1 in cells (Figure 1B). Similar experiments were performed to investigate the Riz1 tumor suppressor protein, which was previously reported to possess H3K9 methyltransferase activity *in vitro* (12). Western analysis of the HA-immunoprecipitated material demonstrated that ectopic full-length Riz1 interacts with HA-PR-Set7 in cells but not with the negative control HA-p53 (Figure 1B). Identical results were obtained following HA-immunoprecipitation of HeLa nuclear lysates expressing HA-PR-Set7 or HA-p53 incubated with *in vitro* translated ^35^S-Riz1 (Figure 1C). *In vitro* binding assays demonstrated that PR-Set7 directly binds Riz1 but not the negative control G9a (Figure 1D). These collective findings indicate that PR-Set7 specifically and directly binds the Riz1 H3K9 methyltransferase *in vitro* and in cells.

### PR-Set7 and Riz1 directly bind via their C-terminal domains

**Figure 2.**
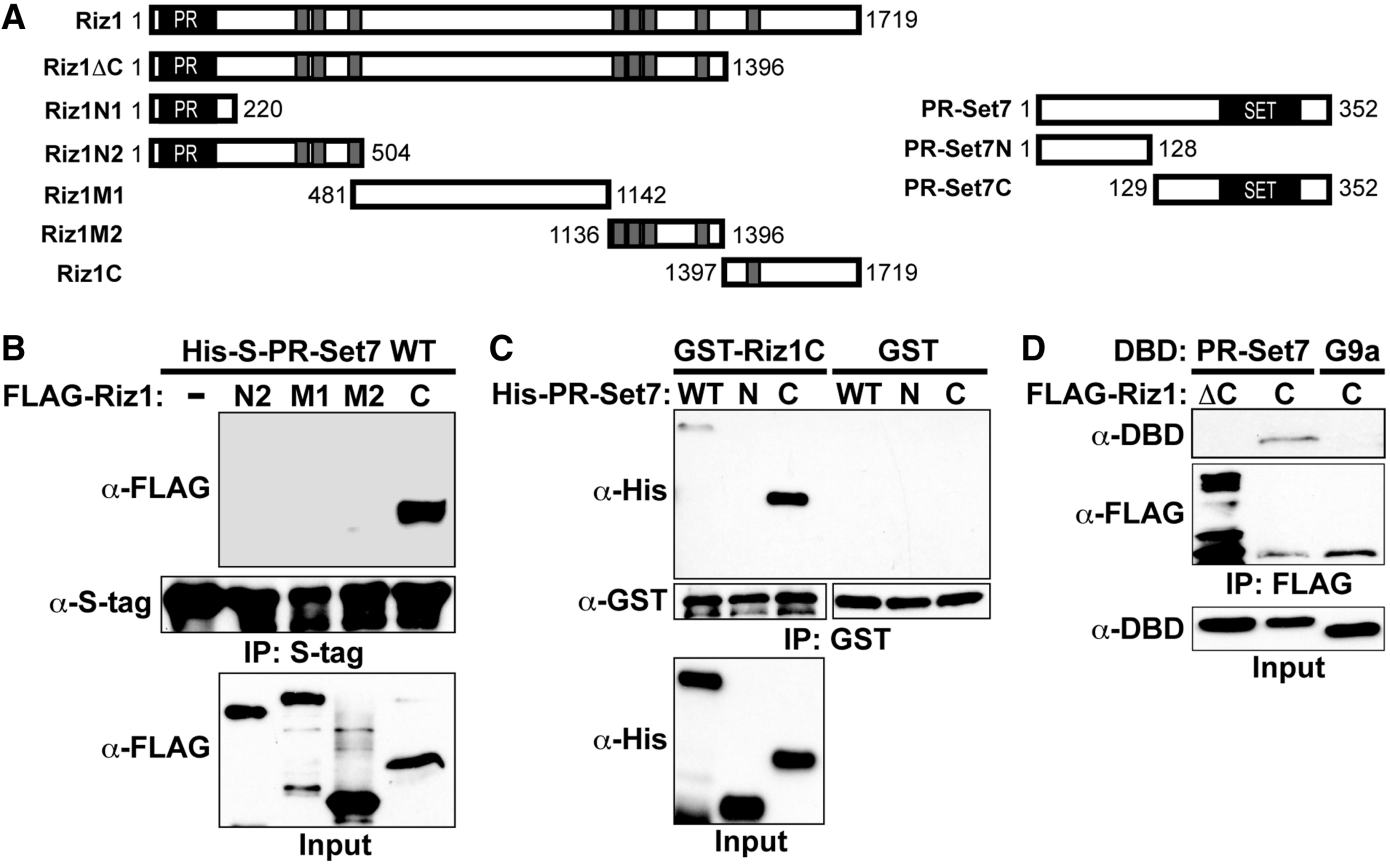
The C-terminal domains are required for Riz1-PR-Set7 binding. (**A**) Illustrations of recombinant Riz1 and PR-Set7 proteins used in the experiments. The catalytic PR/SET domains (black) and zinc fingers (gray) are denoted. (**B**) The indicated recombinant FLAG-Riz1 proteins were incubated *in vitro* with recombinant His-S-PR-Set7 before S-tag-immunoprecipitation and Western analysis. (**C**) The indicated recombinant His-PR-Set7 truncated proteins were incubated *in vitro* with recombinant GST-Riz1C or GST alone before GST pull-down and Western analysis. (**D**) HeLa cells were co-transfected with FLAG-Riz1C or FLAG-Riz1ΔC and DBD-PR-Set7 or DBD-G9a SET domain before FLAG-immunoprecipitation and Western analysis.

To define the minimal Riz1 protein domain required for binding PR-Set7, various recombinant truncations of FLAG-Riz1 were created, purified and incubated *in vitro* with recombinant His-S-PR-Set7 before S-tag-immunoprecipitation (Figure 2A). Western analysis revealed that only the Riz1 C-terminus (aa1397–1719) conferred binding to PR-Set7 (Figure 2B). Similar experiments were performed using recombinant truncations of His-PR-Set7 to define the minimal domain required for binding the GST-Riz1 C-terminus (Riz1C) (Figure 2A). Western analysis following GST-pull down revealed that the C-terminal portion of PR-Set7 containing the catalytic SET domain specifically bound Riz1C (Figure 2C). GST-Riz1C displayed a stronger affinity for His-PR-Set7C compared with wild-type His-PR-Set7 for unknown reasons. It was previously reported that a fragment of Riz1 (aa1514–1680) was sufficient for its homo-dimeric binding to the PR/SET domain (13), suggesting that this region may non-selectively bind any SET domain-containing protein. To test this possibility, HeLa cells were co-transfected with FLAG-Riz1C and DBD-PR-Set7 or DBD-G9aC (SET domain). Western analysis following FLAG-immunoprecipitation demonstrated that Riz1C selectively bound PR-Set7 but not the G9a SET domain (Figure 2D). Importantly, FLAG-Riz1ΔC (aa1–1396) failed to interact with PR-Set7 in these experiments. These results indicate that the C-terminus of Riz1 is required for the selective and direct binding to PR-Set7 *in vitro* and in cells.

### Riz1 is an H3K9 monomethyltransferase

**Figure 3.**
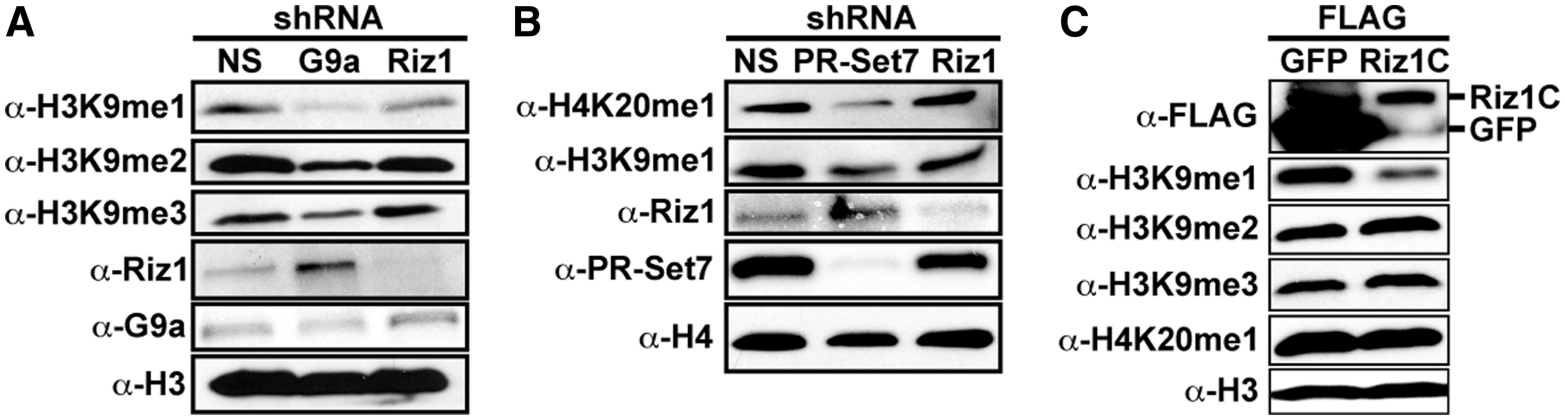
Riz1 is an H3K9 monomethyltransferase*.* (**A**) Western analysis of HeLa cells transfected with non-specific control (NS), Riz1-specific or G9a-specific shRNA plasmids using the indicated antibodies. (**B**) Western analysis of HeLa cells transfected with non-specific control (NS), PR-Set7-specific of Riz1-specific shRNA plasmids using the indicated antibodies. (**C**) Western analysis of HeLa cells transfected with a FLAG-Riz1C or a FLAG-GFP negative control plasmid using the indicated antibodies.

It was previously reported that Riz1 can methylate H3K9 *in vitro*; however, the degree to which Riz1 methylates H3K9 (mono-, di- or trimethyl) remained unknown (12). To address this, HeLa cells were transfected with a control shRNA plasmid or shRNA plasmids that specifically deplete either Riz1 or G9a. Western analysis confirmed that reduction of G9a resulted in the global decrease of all H3K9 methylated forms as previously observed (Figure 3A) (14). Compared with G9a, depletion of Riz1 resulted in a modest, but reproducible, decrease in global H3K9me1 but no significant detectable changes in H3K9me2/me3, demonstrating that Riz1 predominantly functions as an H3K9 monomethyltransferase in cells. It is interesting to note that while PR-Set7 depletion resulted in reduced global H3K9me1 levels, Riz1 depletion did not affect global H4K20me1 levels indicating that Riz1 is dispensable for PR-Set7-mediated H4K20me1 (Figure 3B). Methyltransferase assays confirmed that recombinant Riz1 can mono- and dimethylate K9 of histone H3 synthetic peptide substrates *in vitro* (Supplementary Figure S2). These collective results demonstrate that Riz1 is an H3K9 mono- and dimethyltransferase *in vitro*, that Riz1 functions predominantly as an H3K9 monomethyltransferase in cells and suggests that Riz1-mediated H3K9me1 in cells requires binding to PR-Set7.

The findings above predict that inhibiting Riz1 from binding PR-Set7 would prevent Riz1-mediated H3K9me1. Based on this, we hypothesized that ectopic expression of the PR-Set7 binding domain would cause a dominant negative phenotype by competitively inhibiting endogenous Riz1 binding and resulting in decreased cellular H3K9me1. Consistent with the hypothesis, HeLa cells expressing FLAG-Riz1C displayed a specific and significant reduction of H3K9me1 compared with cells expressing the FLAG-GFP negative control (Figure 3C). It is important to note that the probable binding of FLAG-Riz1C to the catalytic SET domain of PR-Set7 did not inhibit endogenous PR-Set7 enzymatic activity, as no changes in H4K20me1 levels were observed. These findings demonstrate the functional requirement and importance of the PR-Set7 binding domain in Riz1-mediated H3K9me1.

### The PR-Set7 binding domain is necessary for proper nuclear localization of Riz1

**Figure 4.**
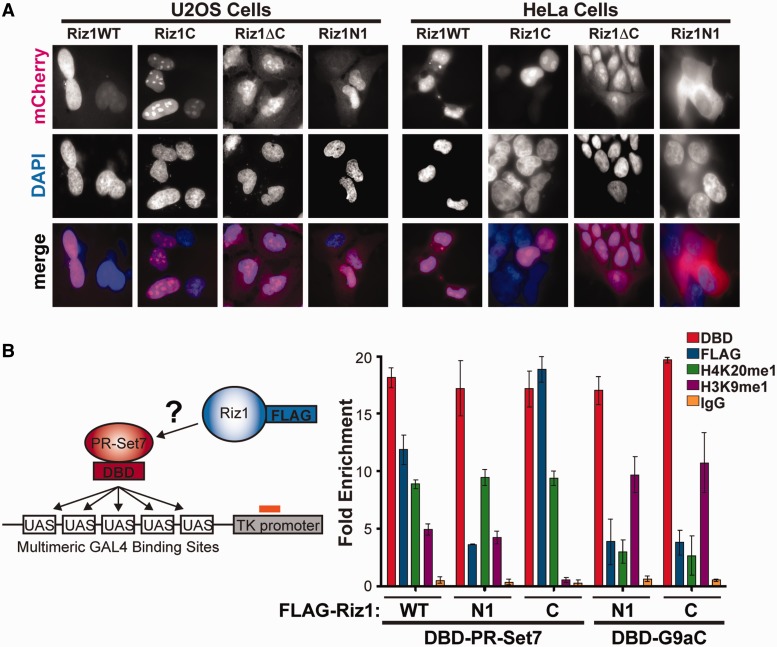
The PR-Set7 binding domain is required for Riz1 nuclear localization and recruitment to chromatin. (**A**) Fluorescence microscopy of U2OS (left) and HeLa (right) cells transfected with the indicated mCherry-Riz1 plasmids (red) and counter-stained with DAPI (blue). (**B**) Illustration of the 5xGAL4-UAS-TK-Luc integrated transgene in HEK293-TK22 cells. A GAL4-DBD-PR-Set7 or GAL4-DBD-G9aC (SET domain) negative control plasmid was co-transfected with the indicated FLAG-Riz1 plasmids in HEK293-TK22 cells (x-axis). ChIPs were performed with the indicated antibodies, and qPCR of the ChIP-DNA was used to determine the fold enrichment at the TK promoter relative to the input DNA (y-axis). The averages and standard error of three independent biological replicates are plotted.

Based on the results above, we hypothesized that the PR-Set7 binding domain (Riz1C) was required for the normal cellular distribution of Riz1. To investigate cellular distribution, mCherry plasmids fused to full-length Riz1 (WT), Riz1C, Riz1ΔC or Riz1N1 (Figure 2A) were expressed in U2OS cells, fixed, counterstained with DAPI and visualized by fluorescence microscopy. The results show that the majority of Riz1 WT was localized to the nucleus, as previously reported (Figure 4A) (15). Consistent with the hypothesis, Riz1C was detected almost exclusively in the nucleus indicating that the PR-Set7 binding domain of Riz1 is sufficient for nuclear localization. Contrary to the hypothesis, Riz1ΔC was detected in the nucleus; however, a significant amount of Riz1ΔC was also detected in the cytosol indicating that the PR-Set7 binding domain is necessary for complete localization of Riz1 to the nucleus. Similarly, Riz1N1 was detected in both nuclear and cytosolic compartments but, in some cells, was excluded from the nucleus indicating that the PR/SET domain is not sufficient or necessary for Riz1 nuclear localization. Similar results in HeLa cells suggest that nuclear localization of Riz1, facilitated in large part by the PR-Set7 binding domain, is likely conserved across cell types (Figure 4A).

### PR-Set7-dependent recruitment of Riz1 to a specific locus

The findings above suggest that PR-Set7 binding is required for Riz1 recruitment to specific genomic loci. To determine whether PR-Set7 is sufficient to recruit Riz1 to a defined locus, the HEK293-TK22 cell line containing an integrated 5× GAL4-UAS-TK-Luc reporter transgene was used (Figure 4B) (8). A GAL4-DBD-PR-Set7 or a negative control GAL4-DBD-G9aC (SET domain) plasmid was co-transfected with FLAG-Riz1 WT, FLAG-Riz1N1 or FLAG-Riz1C. As predicted, chromatin immunoprecipitations (ChIPs) demonstrated that GAL4-DBD-PR-Set7 and GAL4-DBD-G9aC were enriched at the transgene but not at the FKBP5 negative control gene (Figure 4B, Supplementary Figure S3). Consistent with the data, chromatin-bound GAL4-DBD-PR-Set7, but not GAL4-DBD-G9aC, recruited both FLAG-Riz1 WT and FLAG-Riz1C to the transgene, whereas FLAG-RizN1 was not recruited. As expected, elevated H4K20me1 enrichment was observed at the transgene in cells expressing GAL4-DBD-PR-Set7 compared with GAL4-DBD-G9aC. Enrichment of H3K9me1 was also detected at the transgene in cells expressing GAL4-DBD-PR-Set7 and FLAG-Riz1 WT, but at lower levels compared with the GAL4-DBD-G9aC positive control. Although an interaction between PR-Set7 and Riz1N was not detected (Figure 2B and D), similar levels of H3K9me1 enrichment were unexpectedly observed in cells expressing GAL4-DBD-PR-Set7 and FLAG-Riz1N1 compared with FLAG-Riz1 WT. These findings suggest that endogenous Riz1 effectively binds GAL4-DBD-PR-Set7 resulting in H3K9me1 at the transgene. Consistent with this, cells expressing GAL4-DBD-PR-Set7 and FLAG-Riz1C, which likely competitively inhibits binding of endogenous Riz1 to PR-Set7 (Figure 3C), resulted in the ablation of H3K9me1 at the transgene. These results demonstrate that PR-Set7 is sufficient for Riz1 recruitment and establishment of the H4K20me1-H3K9me1 *trans*-tail ‘histone code’ at an ectopic locus and that Riz1 recruitment to chromatin is mediated by the PR-Set7 binding domain.

### Riz1 is required for the H4K20me1-H3K9me1 *trans*-tail ‘histone code’ but dispensable for gene repression

**Figure 5.**
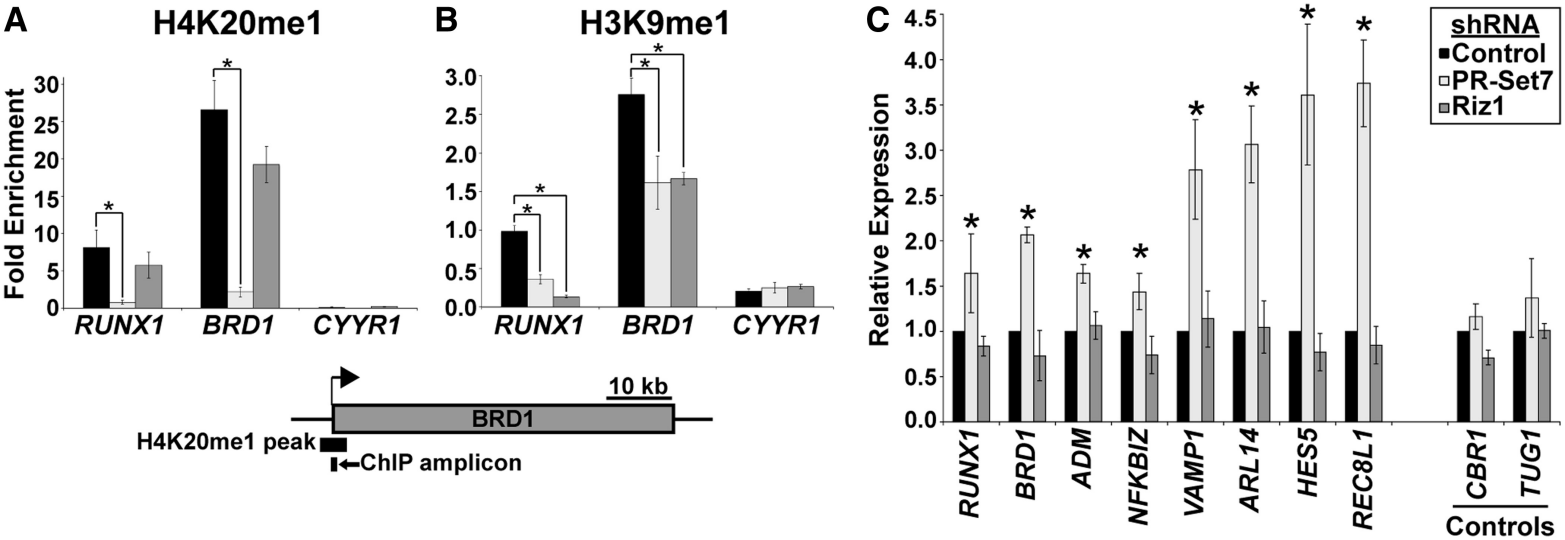
Riz1 is required for the H4K20me1-H3K9me1 trans-tail histone code. ChIPs performed on HeLa cells transfected with non-specific control (black), PR-Set7-specific (light gray) or Riz1-specific (dark gray) shRNAs for H4K20me1 (**A**) or H3K9me1 (**B**). qPCR was performed at an H4K20me1-positive region within the indicated gene (x-axis), as illustrated for *BRD1* (bottom), and results plotted as the fold enrichment relative to input DNA control and histone H3 ChIP control (y-axis). The Student’s *t*-test was used to determine statistically significant changes (*P < 0.05) from three independent biological replicates. (**C**) RT-qPCR analysis of the indicated H4K20me1-positive genes or H4K20me1-negative control genes (x-axis) from HeLa cells transfected with non-specific control (black), PR-Set7-specific (light gray) or Riz1-specific (dark gray) shRNAs. Results were normalized to *18S* expression and plotted relative to non-specific control shRNA (y-axis). The Student’s *t*-test was used to determine statistically significant changes (*P < 0.05) from three independent biological replicates.

To further investigate the interplay between PR-Set7 and Riz1 at endogenous loci, we capitalized on our previous findings that several genes in HeLa cells are enriched for both H4K20me1 and H3K9me1 (4,16). ChIPs at these loci confirmed overlapping enrichment of H4K20me1 and H3K9me1 at *RUNX1* and *BRD1*, but both modifications were absent at the *CYYR1* negative control gene (Figure 5A and B). Depletion of PR-Set7 resulted in the near ablation of H4K20me1 at *RUNX1* and *BRD1*, as previously reported (Figure 5A). The absence of PR-Set7 also resulted in the significant reduction of H3K9me1 at *RUNX1* and *BRD1* (Figure 5B), consistent with the observed global reduction of H3K9me1 (Figure 1A). Depletion of Riz1 led to a slight, but not statistically significant, reduction of H4K20me1 at *RUNX1* and *BRD1* (Figure 5A), consistent with results demonstrating that Riz1 does not affect global H4K20me1 levels (Figure 3B). The absence of Riz1 resulted in the significant reduction of H3K9me1 at *RUNX1* and *BRD1* to levels similar to or lower than those observed in PR-Set7-depleted cells (Figure 5B). These findings indicate that Riz1 is required for the H3K9me1 observed at endogenous H4K20me1-enriched loci.

Similar to PR-Set7, it was previously reported that Riz1 functions as a transcriptional repressor (17). Therefore, we hypothesized that PR-Set7 and Riz1 generate an H4K20me1-H3K9me1 *trans*-tail ‘histone code’ and function cooperatively to repress gene transcription. To test this, RNA was isolated from HeLa cells expressing a control, a PR-Set7-specific or a Riz1-specific shRNA plasmid, and RT-qPCR was performed on genes repressed by PR-Set7 and H4K20me1 (5,16). Consistent with previous reports, depletion of PR-Set7 resulted in the significant de-repression of H4K20me1-associated genes but did not affect expression of the negative control H4K20me3-associated genes (Figure 5C). Contrary to the hypothesis, the depletion of Riz1 and global reduction of H3K9me1 had no significant effect on the expression of H4K20me1-associated genes examined, including *RUNX1* and *BRD1.* These findings indicate that PR-Set7 and PR-Set7-mediated H4K20me1 are required for the repression of specific genes, whereas the concurrent Riz1-mediated H3K9me1 at these genes is dispensable for repression.

### The PR-Set7 binding domain is necessary for Riz1 tumor suppressor function

**Figure 6.**
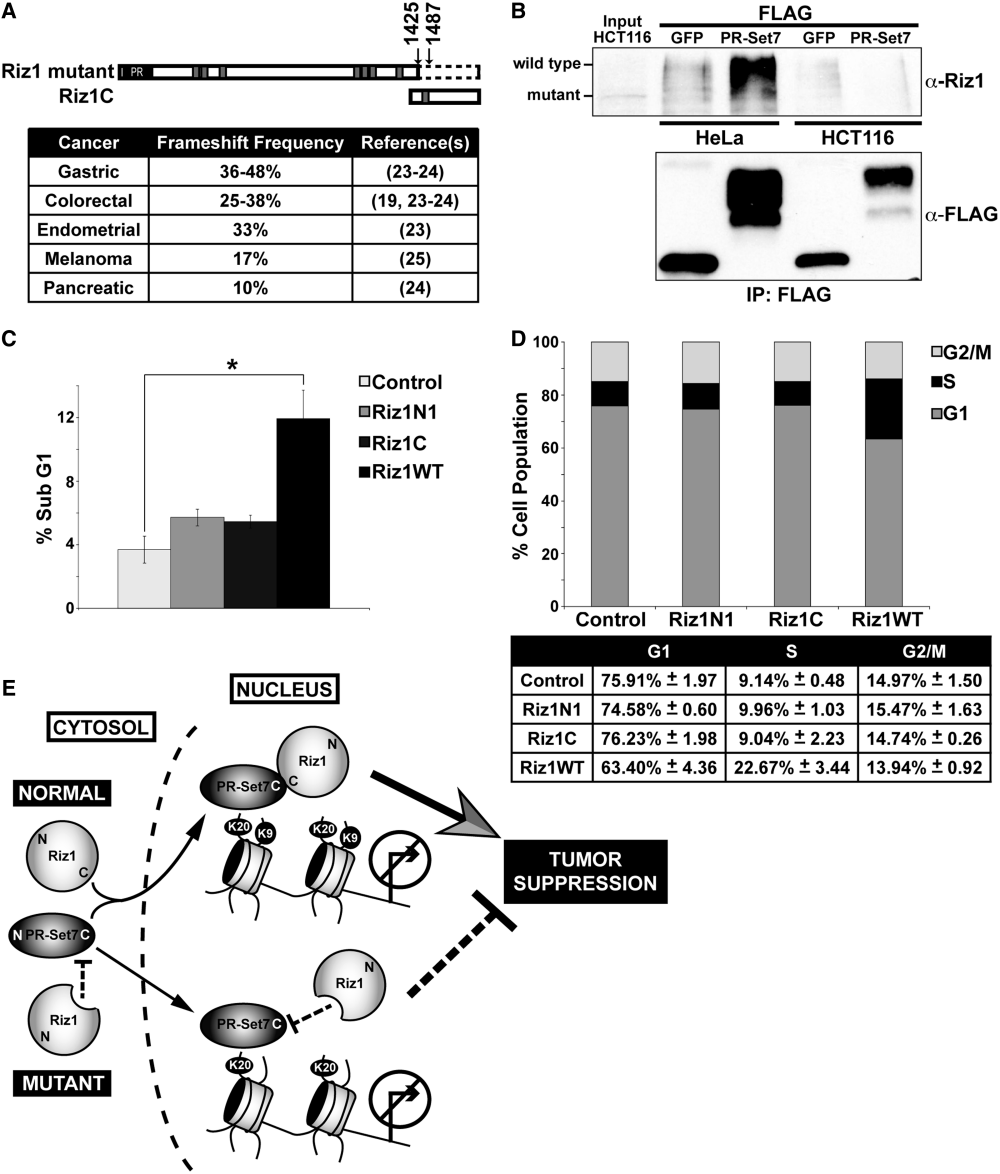
The PR-Set7 binding domain is required for Riz1 tumor suppressor activity. (**A**) Illustration (top) and frequency of *Riz1* poly(A)-tract frameshift mutations in human cancers (bottom) resulting in a truncated Riz1 protein lacking the PR-Set7 binding domain (Riz1C). (**B**) Western analysis using the indicated antibodies following FLAG-immunoprecipitations of HeLa (left) or HCT116 (right) cells transfected with a FLAG-PR-Set7 or a FLAG-GFP negative control plasmid. Truncated mutant Riz1 was detected in HCT116 input (10%). (**C**) HCT116 cells transfected with a control, Riz1 WT, RizN1 or Riz1C mCherry plasmid were stained with Hoechst 33342 before flow cytometry analysis of mCherry-positive cells. The percentage of mCherry-positive cells with sub-G1 content (y-axis) from each sample (x-axis) was determined from three independent biological replicates. A paired Student’s *t* test was used to determine statistical significance (*P < 0.05) compared with control. (**D**) Cell cycle distribution of viable mCherry-positive cells in (C). (**E**) Proposed temporal model based on the findings. The PR-Set7 binding domain of Riz1 (C-terminal) binds the SET domain of PR-Set7 in the cytosol and is required for complete translocation of Riz1 to the nucleus. PR-Set7 recruits Riz1 to specific loci to establish the H4K20me1-H3K9me1 *trans*-tail ‘histone code’. Although Riz1 and H3K9me1 are dispensable for repression of H4K20me1-positive genes, the PR-Set7 binding domain and methyltransferase activity of Riz1 are both required for Riz1 tumor suppressor function.

Previous reports demonstrated that Riz1 functions as a tumor suppressor, as *Riz1*−/− mice develop a wide variety of tumors (18) and transduction of *Riz1* in cancer cells resulted in delayed cell cycle progression and apoptosis (19–21). Consistent with this, loss of heterozygosity of *Riz1* by deletion or promoter hypermethylation is often observed in many different human cancers (18–22). In micro-satellite instable (MSI+) cancers, frameshift mutations in two poly-A tracts of exon 8 of *Riz1* occur frequently yielding a mutant truncated Riz1 protein (Figure 6A) (19,23–25). Because this region encompasses the newly identified PR-Set7 binding domain, we predicted that expression of wild-type Riz1 in cancer cells lacking the Riz1 C-terminal would restore Riz1 tumor suppressor function by delaying cell cycle progression and inducing apoptosis. To test this hypothesis, the HCT116 colorectal cancer cell line was used, as one allele contains a frameshift mutation resulting in a truncated Riz1 while the other allele is silenced by DNA methylation (19). First, HCT116 or HeLa cells were transfected with a FLAG-PR-Set7 or a FLAG-GFP negative control plasmid. Western analysis following FLAG-immunoprecipitations confirmed that endogenous wild-type Riz1 selectively bound PR-Set7 in HeLa cells (Figure 6B). Although a lower molecular weight Riz1 protein was detected in HCT116 lysates, consistent with the reported C-terminal truncation, binding of the endogenous mutant Riz1 to PR-Set7 was not detected. HCT116 cells were then transfected with a control, Riz1 WT, Riz1N1 or Riz1C mCherry plasmid. Flow cytometry of mCherry-positive cells revealed that expression of wild-type Riz1 resulted in a statistically significant 3-fold increase in the number of sub-G1 cells compared with control cells, indicative of apoptosis (Figure 6C). These findings are consistent with previous reports demonstrating significant elevations of apoptotic cells following transduction of *Riz1* in cancer cell lines where *Riz1* is silenced or deleted (19–21). Importantly, significant increases in the number of sub-G1 cells were not observed in Riz1N1- or Riz1C-transfected cells. Cell cycle analysis of viable mCherry-positive cells revealed that wild-type Riz1 expression resulted in a ∼10% reduction of G1-phase cells and concomitant ∼10% elevation of S-phase cells compared with control, indicative of an S-phase delay or arrest (Figure 6D). In contrast to our results, previous reports demonstrated that transduction of *Riz1* induced a G2/M arrest (19–21). Although the reasons for these differences remain unresolved, we confirmed that transfection of wild-type *Riz1* was sufficient to delay cell cycle progression of cancer cells, consistent with the other reports. Furthermore, we found that transfection of Riz1N1 or Riz1C was not sufficient to delay HCT116 cell cycle progression. These collective results indicate that both Riz1 methyltransferase activity and PR-Set7 binding domain are required for effective Riz1 tumor suppressor function in cells.

## DISCUSSION

In this report, we discovered that Riz1 functions as an H3K9 monomethyltransferase required to maintain the H4K20me1-H3K9me1 *trans*-tail ‘histone code’ observed in human cells most likely by direct binding to the PR-Set7 H4K20 monomethyltransferase. Consistent with this, the PR-Set7 binding domain of Riz1 was necessary and sufficient for the PR-Set7-dependent recruitment of Riz1 to a defined locus in cells (Figure 4B). In addition, the PR-Set7 binding domain of Riz1, despite the lack of a nuclear localization signal, was necessary and sufficient for proper Riz1 nuclear localization (Figure 4A). Furthermore, competitive inhibition of the PR-Set7 binding domain resulted in cellular reductions of H3K9me1 consistent with the phenotypes observed following PR-Set7 or Riz1 depletion (Figure 3). These results strongly suggest that Riz1 localization and function require direct binding to PR-Set7. Our results also strongly suggest that PR-Set7 is the primary factor in the pathway that establishes the H4K20me1-H3K9me1 *trans*-tail ‘histone code’, as PR-Set7 was required to maintain H3K9me1 in cells, whereas Riz1 was dispensable for global and local H4K20me1 (Figures 1A, 3C and 5A and B). Based on our collective findings, we propose a temporal model for the establishment of the H4K20me1-H3K9me1 *trans*-tail ‘histone code’, as illustrated in Figure 6E. First, the C-terminus of Riz1 selectively binds the SET domain of PR-Set7 in the cytosol. Next, the PR-Set7-Riz1 complex translocates to the nucleus where PR-Set7 is required for guiding the complex to specific genomic loci. The co-localization of PR-Set7 and Riz1 then results in the establishment of the H4K20me1-H3K9me1 *trans*-tail ‘histone code’ observed at these specific loci. Further investigation will be required to validate and refine this model.

While this report revealed the enzymes and temporal pathway required to establish the H4K20me1-H3K9me1 *trans*-tail ‘histone code’ in cells, the biological significance of these concurrent histone modifications at specific genomic regions remains unclear. Based on previous reports, we hypothesized that the modifications cooperate to regulate gene transcription. We previously demonstrated that catalytically active PR-Set7 was required for the repression of an integrated reporter transgene (26) as well as specific endogenous H4K20me1-H3K9me1-associated genes (16). Similarly, Riz1 was also reported to function as a repressor where the first three zinc finger motifs of Riz1 bind GC-rich Sp-1 elements to repress both the HSV TK and SV40 early promoters (17). Contrary to the hypothesis, we found that both H3K9me1 and Riz1 were dispensable for repression of the genes investigated in this study despite the requirement of Riz1 to maintain the H4K20me1-H3K9me1 *trans*-tail ‘histone code’ at these genes. Therefore, PR-Set7, but not Riz1, is necessary for repression of specific genes, most likely by monomethylating H4K20. PR-Set7 and Riz1 were also reported to function in gene activation, including the induction of estrogen-responsive genes, suggesting a possible role for the H4K20me1-H3K9me1 *trans*-tail ‘histone code’ in these pathways (27,28). Although PR-Set7 and Riz1 primarily function in gene repression, further investigation is required to examine their possible function in gene activation.

In an attempt to elucidate the function of the H4K20me1-H3K9me1 *trans*-tail histone, we reasoned that it should be directly related to other known functions of Riz1 besides gene regulation. Riz1 was originally identified as an Rb-binding protein that functions as a tumor suppressor by inducing cell cycle arrest and apoptosis (18,20). Consistent with its antineoplastic effects, *Riz1* knockout mice develop a variety of tumors and *Riz1* is frequently lost, silenced or mutated in different human cancers including breast, liver and colon cancers and also in neuroblastoma, melanoma, osteosarcoma and malignant meningiomas (18–22). Earlier studies attributed the loss of Riz1 tumor suppressor function to missense mutations in the PR/SET domain (18). However, loss of Riz1 tumor suppressor function is also frequently observed in MSI+ cancers where the PR/SET domain is retained but the C-terminal region is lost due to frameshift mutations in two poly-A tracts of exon 8, including the HCT116 cell line used in this study (19). These findings suggest that the PR/SET domain is required, but not sufficient, for Riz1 tumor suppressor activity and that the C-terminal region has an important, but unknown, role in Riz1 function. We discovered that the Riz1 C-terminal region selectively and directly binds PR-Set7 and that this region was required for Riz1 recruitment to chromatin (Figures 2 and 4). The expression of the Riz1 PR/SET domain or PR-Set7 binding domain alone in HCT116 cells did not rescue Riz1 tumor suppressor function. However, expression of wild-type Riz1 resulted in delayed cell cycle progression and increased apoptosis, comparable with previous reports (19–21), indicating that Riz1 methyltransferase activity and the PR-Set7 binding domain are both necessary for Riz1 tumor suppressor function. Owing to the high frequency of *Riz1* frameshift mutations in various cancers, these findings support the potential clinical utility of *Riz1* gene therapy for the effective treatment of MSI+ cancers (29).

Our results strongly suggest that Riz1 tumor suppressor activity requires Riz1 binding to PR-Set7 and the establishment of the H4K20me1-H3K9me1 *trans*-tail ‘histone code’ at specific loci. Although the precise mechanisms and pathways where Riz1 functions to prevent oncogenesis are unclear, our findings predict that they will coincide with the known tumor suppressive functions of PR-Set7. Recent reports indicate that PR-Set7 and H4K20me1 are central components of the mammalian cell cycle required for proper DNA replication and mitosis (30). Riz1 and H3K9me1 may function cooperatively in DNA replication to form origins of replication and/or regulate firing of the pre-replication complex to prevent aberrant genome duplication (31). Riz1 and H3K9me1 may also function cooperatively with PR-Set7 to ensure proper mitotic chromosome segregation and prevent genomic instability (6). The loss of PR-Set7 was reported to result in persistent DNA double strand breaks suggesting a function for H4K20me1, and possibly Riz1-mediated H3K9me1, in DNA repair (32). Further investigation is required to elucidate the mechanistic functions of Riz1 and the H4K20me1-H3K9me1 *trans*-tail ‘histone code’ in these tumor suppressive pathways.

## SUPPLEMENTARY DATA

Supplementary Data are available at NAR Online.

## FUNDING

National Institutes of Health [GM075094 to J.C.R.]; the American Cancer Society [RSG117619 to J.C.R., PF0627301 to C.T.T.]; the Margaret E. Early Research Trust [to J.C.R.]; the Pew Charitable Trusts [to J.C.R.]; and National Institutes of Health Training Grant [5T32CA009320 to C.T.T.]; The USC Flow Cytometry Core Facility was supported in part by a National Cancer Institute CCSG award [P30CA014089] and the USC Provost Office, Dean’s Development Funds, Keck School of Medicine. Funding for open access charge: American Cancer Society [RSG117619].

*Conflict of interest statement*. None declared.

## Supplementary Material

Supplementary Data
