# Supplementary material for: The PR-Set7 binding domain of Riz1 is required for the H4K20me1-H3K9me1 trans-tail ‘histone code’ and Riz1 tumor suppressor function
Source: Nucleic Acids Res. 2014 Jan 13;42(6):3580–9. doi: 10.1093/nar/gkt1377 (PMC3973283; doi:10.1093/nar/gkt1377)
Supplement: Supplementary Data [file supp_42_6_3580__index.html]

The PR-Set7 binding domain of Riz1 is required for the H4K20me1-H3K9me1 trans-tail ‘histone code’ and Riz1 tumor suppressor function — The PR-Set7 binding domain of Riz1 is required for the H4K20me1-H3K9me1 trans-tail ‘histone code’ and Riz1 tumor suppressor function — Supplementary Data 

# The PR-Set7 binding domain of Riz1 is required for the H4K20me1-H3K9me1 *trans*-tail ‘histone code’ and Riz1 tumor suppressor function

## Supplementary Data

files

**Files in this Data Supplement:**

- Supplementary Data - pdf file
