# Supplementary material for: The PR-Set7 binding domain of Riz1 is required for the H4K20me1-H3K9me1 trans-tail ‘histone code’ and Riz1 tumor suppressor function
Source: Nucleic Acids Res. 2014 Jan 13;42(6):3580–9. doi: 10.1093/nar/gkt1377 (PMC3973283; doi:10.1093/nar/gkt1377)
Supplement: Supplementary Data [file supp_gkt1377_nar-02914-x-2013-File008.pdf]

SUPPLEMENTARY DATA

The PR-Set7 binding domain of Riz1 is required for the
H4K20me1-H3K9me1 *trans*-tail "histone code" and Riz1 tumor suppressor function

Lauren M. Congdon, Jennifer K. Sims, Creighton T. Tuzon and Judd C. Rice

University of Southern California Keck School of Medicine, Department of Biochemistry and Molecular Biology, Harlyne J. Norris Cancer Research Tower, Los Angeles, California 90033, USA

Supplemental Figure S1

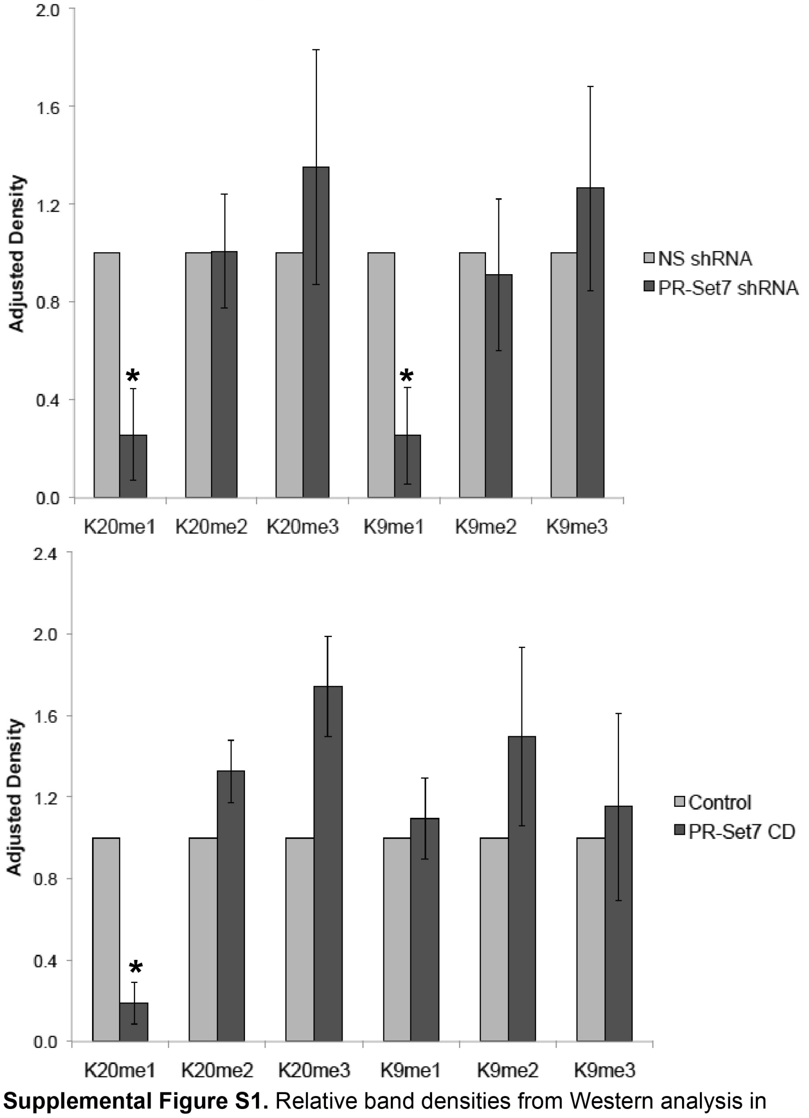

Supplemental Figure S1. Relative band densities from Western analysis in Figure 1A and 2 other independent replicates were quantified using Image J software. Density was normalized to H4 general loading control and plotted relative to NS shRNA or Control samples (grey). The Student t-test was used to determine statistically significant changes ($p<0.03$(*)) from three independent biological replicates.

Supplemental Figure S2

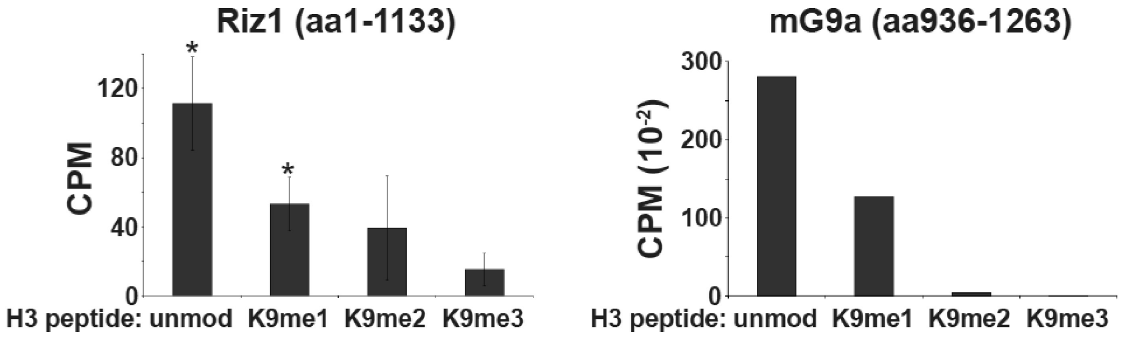

Supplemental Figure S2: *In vitro* histone methyltransferase assays were performed using recombinant Riz1 (left) or mouse G9a (right) proteins. Briefly, 3µg recombinant enzyme was incubated with 1 µg each of the H3K9-methyl peptides and 1 µCi of S-adenosyl-L[methyl-3H]methionine in HMT buffer (final concentration of 25 mM Tris-HCl pH 8.0, 5% glycerol) and incubated at 30°C for 90 minutes. The reaction was spotted on Whatman P-81 paper, washed and counted by liquid scintillation. The indicated values were normalized to reactions lacking peptide substrates.

Supplemental Figure S3

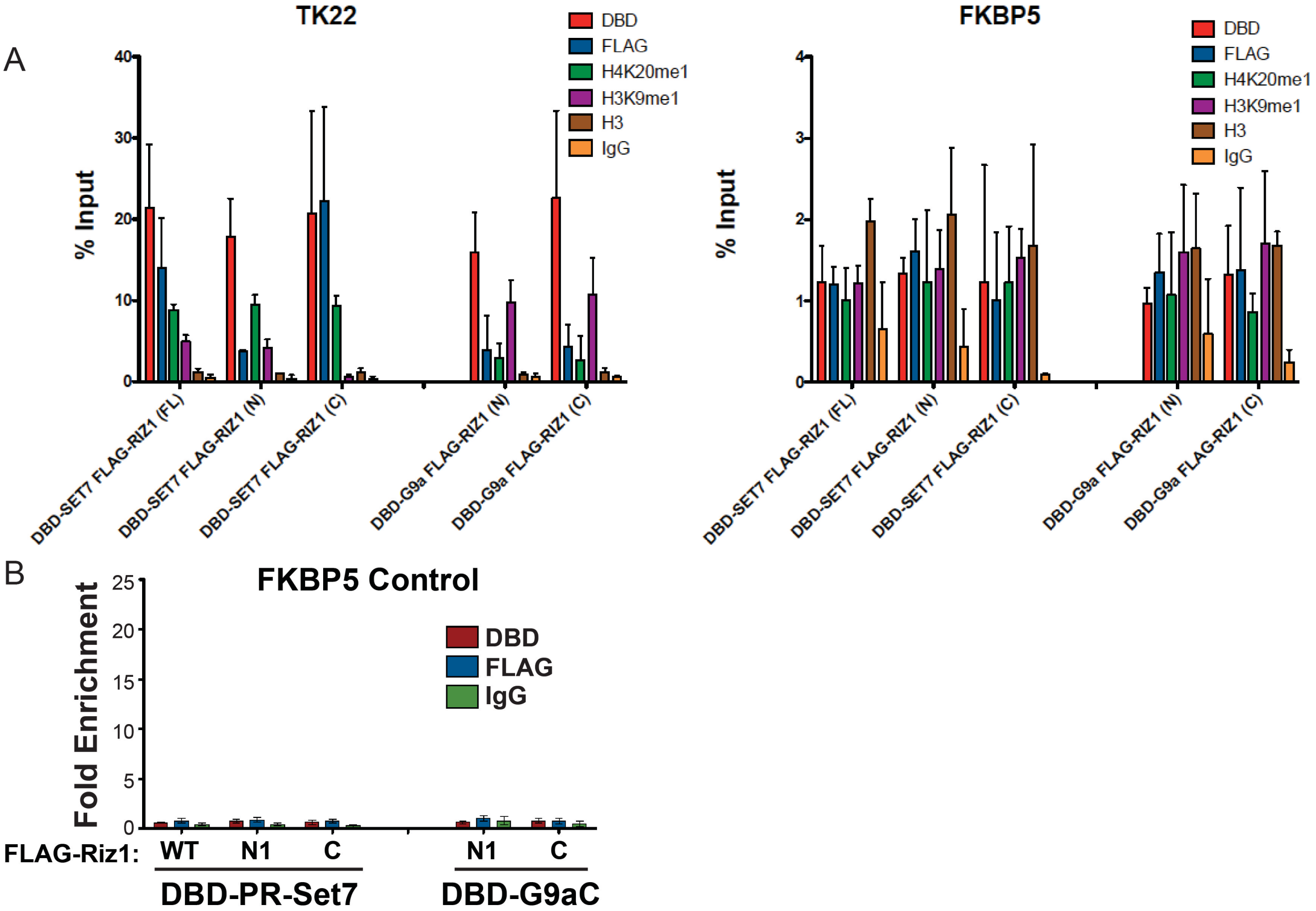

(A) ChIP data from Figure 4C plotted as % Input (2^(Ct Input - Ct IP). A GAL4-DBD-PR-Set7 or GAL4-DBD-G9aC (SET domain) negative control plasmid was co-transfected with the indicated FLAG-Riz1 plasmids in HEK293-TK22 cells (x-axis). ChIPs were performed with the indicated antibodies and qPCR of the ChIP-DNA was used to determine the % of Input at the TK promoter (left) or FKBP5 negative control gene (right). The averages and standard error of three independent biological replicates are plotted. (B) Fold enrichment of ChIPed DNA samples in Figure 4B at the FKBP5 negative control gene relative to the input DNA (y-axis). The averages and standard error of three independent biological replicates are plotted.
